# (η^4^-Cyclo­octa-1,5-diene)diiodidoplatinum(II)

**DOI:** 10.1107/S1600536809029997

**Published:** 2009-08-08

**Authors:** Marie-Hélène Thibault, Frédéric-Georges Fontaine

**Affiliations:** a Département de Chimie, Pavillon Alexandre-Vachon, Local 2257, 1045 Avenue de la Médecine, Université Laval, Québec, Canada G1V 0A6

## Abstract

The monoclinic title complex, [PtI_2_(C_8_H_12_)], characterized by a twisted cyclo­octa­diene ring, is similar to its Cl and Br ortho­rhom­bic homologues. The observed Pt—I bond distances of 2.6094 (5) and 2.6130 (5) Å are in the expected range for PtI_2_ complexes. The C=C double bonds in the mol­ecule differ significantly [1.373 (10) and 1.403 (10) Å]. As expected for a platinum(II) complex, the Pt^II^ atom is in a square-planar environment (ΣPt_α_= 359.71°).

## Related literature

For related structures, see: Thibault *et al.* (2009 ▶); Syed *et al.* (1984 ▶); Wiedermann *et al.* (2005 ▶).
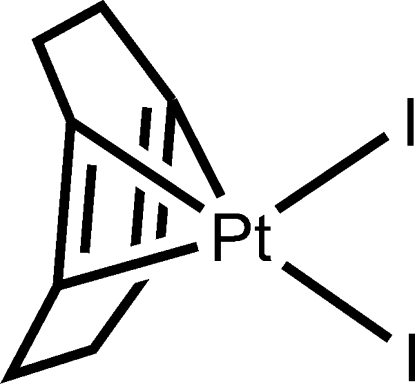


## Experimental

### 

#### Crystal data

[PtI_2_(C_8_H_12_)]
*M* *_r_* = 557.07
Monoclinic, 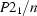

*a* = 8.3063 (13) Å
*b* = 10.8918 (17) Å
*c* = 12.939 (2) Å
β = 106.892 (2)°
*V* = 1120.1 (3) Å^3^
*Z* = 4
Mo *K*α radiation
μ = 17.98 mm^−1^
*T* = 296 K
0.58 × 0.56 × 0.42 mm

#### Data collection

Bruker APEXII CCD diffractometer
Absorption correction: integration (**XPREP**; Bruker, 2005 ▶) *T*_min_ = 0.023, *T*_max_ = 0.049
13155 measured reflections
2714 independent reflections
2488 reflections with *I* > 2σ(*I*)
*R*_int_ = 0.042

#### Refinement

*R*[*F* ^2^ > 2σ(*F* ^2^)] = 0.028
*wR*(*F* ^2^) = 0.073
*S* = 1.09
2714 reflections
105 parameters
H-atom parameters constrained
Δρ_max_ = 2.65 e Å^−3^
Δρ_min_ = −1.77 e Å^−3^

### 

Data collection: *APEX2* (Bruker, 2005 ▶); cell refinement: *SAINT* (Bruker, 2003 ▶); data reduction: *SAINT*; program(s) used to solve structure: *SHELXS97* (Sheldrick, 2008 ▶); program(s) used to refine structure: *SHELXL97* (Sheldrick, 2008 ▶); molecular graphics: *SHELXTL* (Sheldrick, 2008 ▶); software used to prepare material for publication: *SHELXTL*.

## Supplementary Material

Crystal structure: contains datablocks I, global. DOI: 10.1107/S1600536809029997/bg2283sup1.cif

Structure factors: contains datablocks I. DOI: 10.1107/S1600536809029997/bg2283Isup2.hkl

Additional supplementary materials:  crystallographic information; 3D view; checkCIF report

## Figures and Tables

**Fig. 1.**
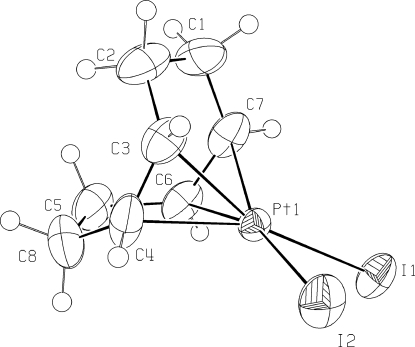
The molecular structure of 1 showing the numbering scheme adopted. Anisotropic atomic displacement ellipsoids for the non-hydrogen atoms are shown at the 50% probability level.

## supplementary crystallographic information

### Comment

The title compound crystallizes in the P2(1)/n space group (Figure 1). Comparison with its dichloro- and dibromo- derivatives shows an important difference as the latter both crystallize in a P2(1)2(1)2(1) space group.

The general aspect of the diiodo complex is similar to the PtCl_2_ (Syed *et al.* 1984) and PtBr_2_ (Wiedermann *et al.* 2005) complexes with a twisted cyclooctadiene ring. Pt—I bond distances of 2.6094 (5) and 2.6130 (5) Å are in the range expected for PtI_2_ complexes. The C=C double bonds C3—C4 and C6—C7 are of significantly different lenghts (1.373 (10) and 1.403 (10) Å respectively). As expected for platinum(II) complexes, the platinum atom is in a square planar environment (ΣPt_α_= 359.71°).

### Experimental

Diiodo(1,5-cyclooctadiene)platinum(II) was purchased from Strem chemicals and used as received. Crystals were grown by slow evaportion of a codPtI_2_ solution in CH_2_Cl_2_.

### Refinement

All hydrogen atoms were placed in idealized position and refined using a riding model with d(C–H) = 0.98 Å, *U*_iso_=1.2*U_eq_* (C) for vinylic protons and 0.97 Å, *U*_iso_=1.2*U*_eq_ (C) for methylene protons.

### Crystal data

**Table:**

[PtI_2_(C_8_H_12_)]	*F*(000) = 976
*M**_r_* = 557.07	*D*_x_ = 3.303 Mg m^−^^3^
Monoclinic, *P*2_1_/*n*	Mo *K*α radiation, λ = 0.71073 Å
Hall symbol: -P 2yn	Cell parameters from 8928 reflections
*a* = 8.3063 (13) Å	θ = 2.5–28.1°
*b* = 10.8918 (17) Å	µ = 17.98 mm^−^^1^
*c* = 12.939 (2) Å	*T* = 296 K
β = 106.892 (2)°	Rectangulaire, yellow
*V* = 1120.1 (3) Å^3^	0.58 × 0.56 × 0.42 mm
*Z* = 4	

### Data collection

**Table:**

Bruker APEXII CCD diffractometer	2714 independent reflections
Radiation source: fine-focus sealed tube	2488 reflections with *I* > 2σ(*I*)
graphite	*R*_int_ = 0.042
ω scans	θ_max_ = 28.1°, θ_min_ = 2.5°
Absorption correction: integration (*XPREP*; Bruker, 2005)	*h* = −10→10
*T*_min_ = 0.023, *T*_max_ = 0.049	*k* = −14→14
13155 measured reflections	*l* = −17→16

### Refinement

**Table:**

Refinement on *F*^2^	Secondary atom site location: difference Fourier map
Least-squares matrix: full	Hydrogen site location: inferred from neighbouring sites
*R*[*F*^2^ > 2σ(*F*^2^)] = 0.028	H-atom parameters constrained
*wR*(*F*^2^) = 0.073	*w* = 1/[σ^2^(*F*_o_^2^) + (0.0427*P*)^2^ + 1.2011*P*] where *P* = (*F*_o_^2^ + 2*F*_c_^2^)/3
*S* = 1.09	(Δ/σ)_max_ = 0.002
2714 reflections	Δρ_max_ = 2.65 e Å^−^^3^
105 parameters	Δρ_min_ = −1.77 e Å^−^^3^
0 restraints	Extinction correction: *SHELXL97* (Sheldrick, 2008), Fc^*^=kFc[1+0.001xFc^2^λ^3^/sin(2θ)]^-1/4^
Primary atom site location: structure-invariant direct methods	Extinction coefficient: 0.00188 (15)

### Special details

**Table:**

Geometry. All e.s.d.'s (except the e.s.d. in the dihedral angle between two l.s. planes) are estimated using the full covariance matrix. The cell e.s.d.'s are taken into account individually in the estimation of e.s.d.'s in distances, angles and torsion angles; correlations between e.s.d.'s in cell parameters are only used when they are defined by crystal symmetry. An approximate (isotropic) treatment of cell e.s.d.'s is used for estimating e.s.d.'s involving l.s. planes.
Refinement. Refinement of *F*^2^ against ALL reflections. The weighted *R*-factor *wR* and goodness of fit *S* are based on *F*^2^, conventional *R*-factors *R* are based on *F*, with *F* set to zero for negative *F*^2^. The threshold expression of *F*^2^ > σ(*F*^2^) is used only for calculating *R*-factors(gt) *etc*. and is not relevant to the choice of reflections for refinement. *R*-factors based on *F*^2^ are statistically about twice as large as those based on *F*, and *R*- factors based on ALL data will be even larger.

### Fractional atomic coordinates and isotropic or equivalent isotropic displacement parameters (Å^2^)

**Table:**

	*x*	*y*	*z*	*U*_iso_*/*U*_eq_	
Pt1	0.24239 (2)	0.111767 (17)	0.248867 (13)	0.02846 (9)	
I1	0.54151 (5)	0.14392 (4)	0.38327 (4)	0.05103 (13)	
I2	0.37225 (5)	0.08198 (4)	0.08927 (3)	0.05224 (13)	
C3	−0.0015 (7)	0.0311 (6)	0.1590 (5)	0.0481 (13)	
H3	0.0045	−0.0247	0.1008	0.07 (2)*	
C2	−0.0933 (8)	−0.0214 (6)	0.2341 (6)	0.0600 (17)	
H2A	−0.1348	−0.1026	0.2091	0.072*	
H2B	−0.1898	0.0300	0.2316	0.072*	
C6	0.1178 (7)	0.1941 (6)	0.3600 (5)	0.0453 (13)	
H6	0.1898	0.2488	0.4142	0.09 (3)*	
C7	0.1446 (8)	0.0687 (7)	0.3837 (5)	0.0492 (14)	
H7	0.2319	0.0518	0.4515	0.040 (15)*	
C4	−0.0014 (8)	0.1533 (7)	0.1329 (6)	0.0539 (16)	
H4	0.0043	0.1683	0.0594	0.09 (3)*	
C1	0.0170 (9)	−0.0302 (7)	0.3499 (6)	0.0645 (18)	
H1A	−0.0545	−0.0288	0.3973	0.077*	
H1B	0.0746	−0.1087	0.3597	0.077*	
C5	−0.0526 (8)	0.2486 (6)	0.2996 (6)	0.0620 (18)	
H5A	−0.1407	0.1991	0.3142	0.074*	
H5B	−0.0607	0.3306	0.3270	0.074*	
C8	−0.0825 (9)	0.2558 (7)	0.1778 (7)	0.076 (2)	
H8A	−0.2027	0.2544	0.1425	0.091*	
H8B	−0.0394	0.3335	0.1606	0.091*	

### Atomic displacement parameters (Å^2^)

**Table:**

	*U*^11^	*U*^22^	*U*^33^	*U*^12^	*U*^13^	*U*^23^
Pt1	0.02846 (13)	0.02936 (13)	0.02927 (14)	0.00017 (6)	0.01107 (9)	−0.00161 (6)
I1	0.0321 (2)	0.0726 (3)	0.0459 (2)	−0.00714 (15)	0.00754 (17)	−0.00476 (18)
I2	0.0537 (3)	0.0707 (3)	0.0402 (2)	0.00935 (18)	0.02613 (18)	−0.00156 (18)
C3	0.040 (3)	0.056 (3)	0.045 (3)	−0.011 (2)	0.007 (2)	−0.015 (3)
C2	0.056 (4)	0.055 (4)	0.073 (4)	−0.024 (3)	0.025 (3)	−0.020 (3)
C6	0.042 (3)	0.056 (3)	0.045 (3)	−0.006 (2)	0.023 (2)	−0.019 (3)
C7	0.040 (3)	0.083 (4)	0.029 (3)	−0.005 (3)	0.017 (2)	0.001 (3)
C4	0.031 (3)	0.082 (5)	0.047 (4)	0.013 (3)	0.008 (3)	0.008 (3)
C1	0.066 (4)	0.062 (4)	0.075 (5)	−0.012 (3)	0.037 (4)	0.013 (3)
C5	0.045 (3)	0.045 (3)	0.102 (6)	0.003 (2)	0.032 (3)	−0.023 (3)
C8	0.051 (4)	0.076 (5)	0.102 (6)	0.027 (3)	0.022 (4)	0.029 (4)

### Geometric parameters (Å, °)

**Table:**

Pt1—C7	2.179 (5)	C6—C5	1.525 (9)
Pt1—C4	2.188 (6)	C6—H6	0.9800
Pt1—C6	2.193 (5)	C7—C1	1.485 (9)
Pt1—C3	2.205 (5)	C7—H7	0.9800
Pt1—I1	2.6094 (5)	C4—C8	1.505 (10)
Pt1—I2	2.6130 (5)	C4—H4	0.9800
C3—C4	1.373 (10)	C1—H1A	0.9700
C3—C2	1.511 (8)	C1—H1B	0.9700
C3—H3	0.9800	C5—C8	1.525 (11)
C2—C1	1.516 (10)	C5—H5A	0.9700
C2—H2A	0.9700	C5—H5B	0.9700
C2—H2B	0.9700	C8—H8A	0.9700
C6—C7	1.403 (10)	C8—H8B	0.9700
			
C7—Pt1—C4	96.2 (3)	Pt1—C6—H6	114.3
C7—Pt1—C6	37.4 (3)	C6—C7—C1	126.0 (6)
C4—Pt1—C6	81.2 (2)	C6—C7—Pt1	71.8 (3)
C7—Pt1—C3	80.6 (2)	C1—C7—Pt1	108.7 (4)
C4—Pt1—C3	36.4 (3)	C6—C7—H7	114.0
C6—Pt1—C3	88.4 (2)	C1—C7—H7	114.0
C7—Pt1—I1	89.98 (16)	Pt1—C7—H7	114.0
C4—Pt1—I1	160.3 (2)	C3—C4—C8	126.3 (6)
C6—Pt1—I1	92.71 (15)	C3—C4—Pt1	72.4 (3)
C3—Pt1—I1	162.96 (17)	C8—C4—Pt1	108.5 (5)
C7—Pt1—I2	160.3 (2)	C3—C4—H4	113.8
C4—Pt1—I2	89.78 (19)	C8—C4—H4	113.8
C6—Pt1—I2	162.00 (17)	Pt1—C4—H4	113.8
C3—Pt1—I2	93.50 (15)	C7—C1—C2	114.8 (5)
I1—Pt1—I2	90.662 (19)	C7—C1—H1A	108.6
C4—C3—C2	124.1 (6)	C2—C1—H1A	108.6
C4—C3—Pt1	71.1 (3)	C7—C1—H1B	108.6
C2—C3—Pt1	111.7 (4)	C2—C1—H1B	108.6
C4—C3—H3	114.1	H1A—C1—H1B	107.5
C2—C3—H3	114.1	C8—C5—C6	113.4 (5)
Pt1—C3—H3	114.1	C8—C5—H5A	108.9
C3—C2—C1	112.7 (5)	C6—C5—H5A	108.9
C3—C2—H2A	109.0	C8—C5—H5B	108.9
C1—C2—H2A	109.0	C6—C5—H5B	108.9
C3—C2—H2B	109.0	H5A—C5—H5B	107.7
C1—C2—H2B	109.0	C4—C8—C5	113.9 (6)
H2A—C2—H2B	107.8	C4—C8—H8A	108.8
C7—C6—C5	123.8 (5)	C5—C8—H8A	108.8
C7—C6—Pt1	70.7 (3)	C4—C8—H8B	108.8
C5—C6—Pt1	111.6 (4)	C5—C8—H8B	108.8
C7—C6—H6	114.3	H8A—C8—H8B	107.7
C5—C6—H6	114.3		
			
C7—Pt1—C3—C4	114.0 (4)	I2—Pt1—C7—C6	−173.6 (4)
C6—Pt1—C3—C4	77.2 (4)	C4—Pt1—C7—C1	56.1 (5)
I1—Pt1—C3—C4	171.3 (4)	C6—Pt1—C7—C1	122.8 (6)
I2—Pt1—C3—C4	−84.9 (4)	C3—Pt1—C7—C1	23.0 (5)
C7—Pt1—C3—C2	−6.1 (5)	I1—Pt1—C7—C1	−142.7 (5)
C4—Pt1—C3—C2	−120.2 (6)	I2—Pt1—C7—C1	−50.8 (8)
C6—Pt1—C3—C2	−43.0 (5)	C2—C3—C4—C8	3.5 (11)
I1—Pt1—C3—C2	51.1 (8)	Pt1—C3—C4—C8	−100.4 (7)
I2—Pt1—C3—C2	155.0 (4)	C2—C3—C4—Pt1	103.9 (6)
C4—C3—C2—C1	−93.3 (8)	C7—Pt1—C4—C3	−65.0 (4)
Pt1—C3—C2—C1	−12.0 (7)	C6—Pt1—C4—C3	−99.4 (4)
C4—Pt1—C6—C7	112.4 (4)	I1—Pt1—C4—C3	−172.5 (4)
C3—Pt1—C6—C7	76.6 (4)	I2—Pt1—C4—C3	96.2 (4)
I1—Pt1—C6—C7	−86.4 (3)	C7—Pt1—C4—C8	58.2 (5)
I2—Pt1—C6—C7	173.0 (4)	C6—Pt1—C4—C8	23.8 (5)
C7—Pt1—C6—C5	−119.7 (6)	C3—Pt1—C4—C8	123.2 (7)
C4—Pt1—C6—C5	−7.2 (4)	I1—Pt1—C4—C8	−49.2 (9)
C3—Pt1—C6—C5	−43.1 (4)	I2—Pt1—C4—C8	−140.6 (5)
I1—Pt1—C6—C5	153.9 (4)	C6—C7—C1—C2	43.1 (9)
I2—Pt1—C6—C5	53.4 (7)	Pt1—C7—C1—C2	−37.6 (8)
C5—C6—C7—C1	3.3 (9)	C3—C2—C1—C7	33.5 (9)
Pt1—C6—C7—C1	−100.3 (6)	C7—C6—C5—C8	−91.6 (7)
C5—C6—C7—Pt1	103.6 (5)	Pt1—C6—C5—C8	−11.0 (7)
C4—Pt1—C7—C6	−66.7 (4)	C3—C4—C8—C5	43.9 (10)
C3—Pt1—C7—C6	−99.8 (4)	Pt1—C4—C8—C5	−37.5 (8)
I1—Pt1—C7—C6	94.5 (3)	C6—C5—C8—C4	32.8 (9)
